# Endoscopic Management of Postoperative Stapled Anastomosis Bleeding

**DOI:** 10.5005/jp-journals-10018-1100

**Published:** 2014-01-22

**Authors:** Arunkumar Krishnan, Vimalraj Velayutham, Jeswanth Satyanesan

**Affiliations:** 1 Centre for GI Bleed & Institute of Surgical Gastroenterology and Liver Transplantation, Stanley Medical College and Hospital, Chennai Tamil Nadu, India; 2 Centre for GI Bleed & Institute of Surgical Gastroenterology and Liver Transplantation, Stanley Medical College and Hospital, Chennai Tamil Nadu, India; 3 Centre for GI Bleed & Institute of Surgical Gastroenterology and Liver Transplantation, Stanley Medical College and Hospital, Chennai Tamil Nadu, India

**Keywords:** EBL, Anastomosis bleeding, Endoscopy.

## Abstract

**To the Editor:** Knowledge has evolved and the use of staplers in gastrointestinal surgery is now widespread. They are associated with low rates of postoperative complications. Postoperative anastomotic complications with stapling devices are relatively rare, with a reported incidence between 0 and 2.5%.1

**How to cite this article:** Krishnan A, Velayutham V, Satyanesan J. Endoscopic Management of Postoperative Stapled Anastomosis Bleeding. Euroasian J Hepato-Gastroenterol 2014;4(1):62-63.

Knowledge has evolved and the use of staplers in gastrointestinal surgery is now widespread. They are associated with low rates ofpostoperative complications. Postoperative anastomotic complications with stapling devices are relatively rare, with a reported incidence between 0 and 2.5%.^1^ A38-year-old male patient was admitted to our department, because of high colored urine and yellowish discoloration of eyes for 6 months. The patient was otherwise healthy and had no previous history of bleeding disorders. Contrast-enhanced computed tomography (CECT) of abdomen showed a mass in the head of the pancreas. He underwent exploration and trial resection and it was found inoperable; palliative triple bypass was performed with liner stapler. On the 12th postoperative day, bleeding as diagnosed with features of postural hypotension and abdominal discomfort was followed by melena was reported. The patient was readmitted to hospital and required four units of blood transfusion in 48 hours. Because, the clinical picture did not improve, an uppergastrointestinal endoscopy was performed, with easy visualization of the bleeding site at the gastrojejunal stapler anastomotic site. The bleeding stopped after the application of two band ligation. An endoscopy was performed after 48 hours, with no further signs of bleeding. There was no further bleeding during 1 year of follow-up. Postoperative bleeding is a serious adverse effect, which may require additional emergency surgery. Postoperative hemorrhage is defined as significant bleeding (>100 ml/h) in the immediate postoperative period that requires reoperation or hemodynamic resuscitation.^1^ Compared with leakage and stricture, anastomotic bleeding is rare and usually self-limited.^2^ The cause ofanastomotic bleeding after stapling is the presence of mesentery within the staple line prior to division with the stapling instrument.^3^ Endoscopy provides a more direct approach to the anastomotic site and, although it may seem aggressive and possibly even dangerous to perform in the immediate postoperative period, it has been shown to be safe in experimental models.^4^ Endoscopic band ligation devices, commonly used in variceal bleeding have been used to treat nonvariceal causes of bleeding and involve the placement of elastic bands over tissue to produce mechanical compression and tamponade to arrest bleeding by applying a direct pressure in case of minor injuries or by applying ligature when a larger vessel is involved. Anastomotic hemorrhage after palliative triple bypass is a rare complication, but, if it occurs, prompt treatment is needed. Postoperative anastomotic bleeding could be avoided by inspection of the anastomosis and ensuring that no anastomotic bleeding occurs during surgery. Endoscopic therapy should be considered when anastomotic bleeding is diagnosed. Endoscopic band ligation may be safely and effectively used in the early postoperative period to stop unremitting anastomotic hemorrhage. It is very effective for hemorrhage from a stapled anastomosis. Endoscopic procedures are useful for confirmation of bleeding and for therapeutic intervention, and may avoid the need of surgical intervention.
